# Effects of Electronic Health Record Implementation and Barriers to Adoption and Use: A Scoping Review and Qualitative Analysis of the Content

**DOI:** 10.3390/life10120327

**Published:** 2020-12-04

**Authors:** Chen Hsi Tsai, Aboozar Eghdam, Nadia Davoody, Graham Wright, Stephen Flowerday, Sabine Koch

**Affiliations:** 1 Health Informatics Centre, Department of Learning, Informatics, Management and Ethics, Karolinska Institutet, 171 77 Stockholm, Sweden; chenhsitsai@outlook.com (C.H.T.); aboozar.eghdam@gmail.com (A.E.); nadia.davoody@ki.se (N.D.); 2 Department of Information Systems, Rhodes University, Grahamstown 6140, South Africa; G.Wright@ru.ac.za (G.W.); s.flowerday@ru.ac.za (S.F.)

**Keywords:** electronic health record, personal health record, scoping review, implementation, adoption

## Abstract

Despite the great advances in the field of electronic health records (EHRs) over the past 25 years, implementation and adoption challenges persist, and the benefits realized remain below expectations. This scoping review aimed to present current knowledge about the effects of EHR implementation and the barriers to EHR adoption and use. A literature search was conducted in PubMed, Web of Science, IEEE Xplore Digital Library and ACM Digital Library for studies published between January 2005 and May 2020. In total, 7641 studies were identified of which 142 met the criteria and attained the consensus of all researchers on inclusion. Most studies (n = 91) were published between 2017 and 2019 and 81 studies had the United States as the country of origin. Both positive and negative effects of EHR implementation were identified, relating to clinical work, data and information, patient care and economic impact. Resource constraints, poor/insufficient training and technical/educational support for users, as well as poor literacy and skills in technology were the identified barriers to adoption and use that occurred frequently. Although this review did not conduct a quality analysis of the included papers, the lack of uniformity in the use of EHR definitions and detailed contextual information concerning the study settings could be observed.

## 1. Introduction

In the early 1990s, a trend in the shift from paper-based health records to electronic records started; this was in response to advances in technology as well as the advocacy of the Institute of Medicine in the United States [1,2]. As a result of the inadequacies of paper-based health records gradually becoming evident to the healthcare industry [3], electronic records have continued to be developed and envisioned with many expected benefits over the past 25 years.

Over those 25 years, the names and terms used to represent the concept of electronic records have changed frequently while the basic idea has remained the same [4]. Nowadays, the term “electronic health record” (EHR) is widely used for records adopted by clinicians [4]. This usage does not, however, comply with the way different types of electronic records have been defined by the International Organization for Standardization (ISO). 

According to ISO/TR 14639-1:2012(en), an “electronic medical record” (EMR) is defined as an “electronic record of an individual in a physician’s office or clinic, which is typically in one setting and is provider-centric”, whereas an “electronic patient record” (EPR) is defined as an “electronic record of an individual in a hospital or health care facility, which is typically in one organization and is facility-centric” [5]. Given the previous two definitions, an electronic health record (EHR) is defined as follows:

“Information relevant to the wellness, health and healthcare of an individual, in computer-processable form and represented according to a standardized information model, or the longitudinal electronic record of an individual that contains or virtually interlines to data in multiple EMRs and EPRs, which is to be shared and/or interoperable across healthcare settings and is patient-centric.”

[5]

Furthermore, a personal health record (PHR) is defined by ISO/TR 14292:2012(en) as

“…a representation of information regarding, or relevant to, the health, including wellness, development and welfare of that individual, which may be stand-alone or may integrate health information from multiple sources, and for which the individual, or the representative to whom the individual delegated his or her rights, manages and controls the PHR content and grants permissions for access by, and/or sharing with, other parties.”

[6]

However, a continuum exists in many countries between the two strict views of the EHR and PHR on the one hand, regarding the entity that has control over the record and the content within it, and the tethered PHRs on the other. In the latter case, the patient is given access to the EHR by the care provider without the patient controlling it. This access function is often part of a patient portal.

Approximately 25 years after the emergence of EHRs, substantial progress has been made regarding EHR implementation, adoption and use [2]. Unfortunately, this has mostly been in an uncoordinated way rather than with a coordinated and logical approach. Many of the initial expectations regarding time efficiency, productivity, and increased quality of care have not been met or have only been partially realized, and “current EHRs still do not meet the needs of today’s rapidly changing healthcare environment” [2]. Data duplication is still a prevailing issue and solutions are still sought even though this was expected to be solved by the uptake of EHRs [7,8,9]. Only recently has there been any significant progress in the development of legal frameworks for patient privacy and confidentiality concerning EHR data [2,10,11]. Continuing progress on standards for EHR data has strengthened the capability of data exchange, the secondary use of data and decision support [2,12]. 

Despite the apparent progress in implementation methods and the use of EHRs, the realization of benefits still lags behind expectations. Great challenges for clinicians as end users of EHRs exist, which restricts their potential to facilitate both the work of clinicians and the improvement of patient care quality [13]. Whether the use of EHRs improves efficiency (i.e., “saves time”) for clinicians or not is still regarded as controversial [2]. While some believe that the adoption of EHRs has improved patient care, further work needs to be undertaken. In particular, identification of the complex mechanism behind the measurement of patient outcomes related to the implementation of EHRs is needed to reach a more concrete conclusion [14]. 

The aim of the study is, therefore, to review the existing literature and elicit current knowledge on the effects of EHR implementation and the barriers to EHR adoption and use.

## 2. Materials and Methods 

In line with Peters et al. [15], a scoping review of the literature without assessing the quality of the included studies was conducted. 

### 2.1. Study Retrieval

Searches were conducted in PubMed, Web of Science, IEEE Xplore, and ACM Digital Library. A comprehensive search strategy was developed where search terms were combined and used in two different sets (set 1: electronic health record, EHR, personal health record, PHR, and patient record; and care pathways, workflow, work routines, workload, and work process; set 2: electronic health record, EHR, personal health record, PHR, and patient record; and efficiency, advantages, disadvantages, satisfaction, teamwork, collaboration, benefits, and challenges) when retrieving the studies. See Table 1 for electronic search strategy.

In total, 8114 studies were identified of which 473 were duplicates that were removed, resulting in 7641 unique and potentially relevant studies.

### 2.2. Study Selection

**Figure 1:**
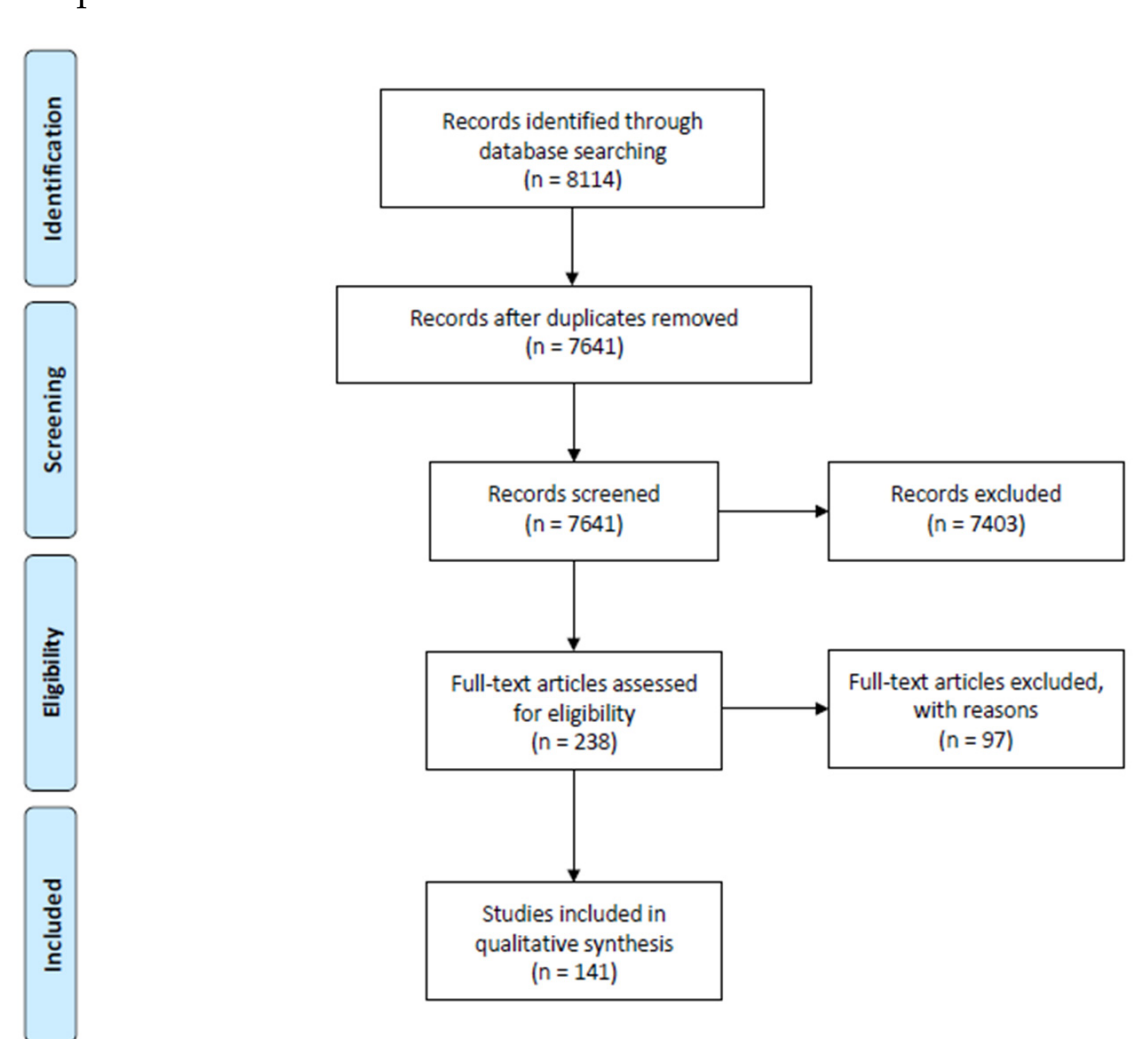
Preferred Reporting Items for Systematic Reviews and Meta-Analyses (PRISMA) flow diagram of the study selection process.

The titles and abstracts of the 7641 studies were manually screened against the inclusion and exclusion criteria. Inclusion criteria were review articles, conference papers and original articles published in English between January 2005 and May 2020, focusing on the barriers to and effects of implementing EHRs or tethered PHRs. Individual studies that were also included in a literature review were not removed. Studies reporting on the effects of implementing tethered PHRs were included as we considered them to be part of EHRs. Exclusion criteria included studies related to secondary use of EHRs, data mining of EHRs, methods for evaluating EHR implementation, and EHR-integrated applications/software/tools. Subsequently, 7403 studies were excluded based on these criteria. This left 238 articles, which were read in full by four researchers (A.E., C.H.T., G.W., and S.K.). Two additional researchers (S.F. and S.K.) were called in for a discussion on the disagreements when comparing the assessments of eligibility. Finally, consensus was reached among all researchers on the inclusion of 141 articles in the final analysis of this scoping review (Figure 1). Full-text articles were excluded with reasons, including meeting the exclusion criteria; investigating partial components of EHRs (e.g., e-prescription and decision support); focusing on system development models/methods, strategic/design recommendations, design prototypes, and usability principles; reporting speculations about success factors, prevalence of use, user group characteristics and differences, workflows, and processes of implementation.

### 2.3. Data Analysis

The full-text pdf files of the 141 studies were imported into NVivo 12. Using the tool, both qualitative and quantitative studies and their results were analyzed qualitatively by adopting a thematic analysis approach [16]. G.W. initially read all the articles, coded/annotated them qualitatively and identified potential themes/categories. C.H.T. performed the same steps independently and then re-read all the articles, reviewed the extracted codes and compared the potential themes/categories created by the two researchers to identify recurrent themes/categories. The final themes/categories were defined clearly for further analysis and reporting of the results after reviewing by S.F. and S.K.

## 3. Results

The majority of the 141 studies were published between 2017 and 2019. The USA was the country of origin for 81 studies and European countries for six studies. Questionnaire/survey (n = 63), interview (n = 33), observation (n = 16) and time-motion observation (n = 7) were some of the common methods used. Study participants were mainly physicians followed by registered nurses/nurse practitioners (Table 2).

The three main themes identified were positive effects, negative effects, and barriers, as shown in Table 3. In the following paragraphs, the identified categories are used as headings to present the combined positive and negative effects and the barriers.

### 3.1. Effects of EHR Implementation

**Figure 2:**
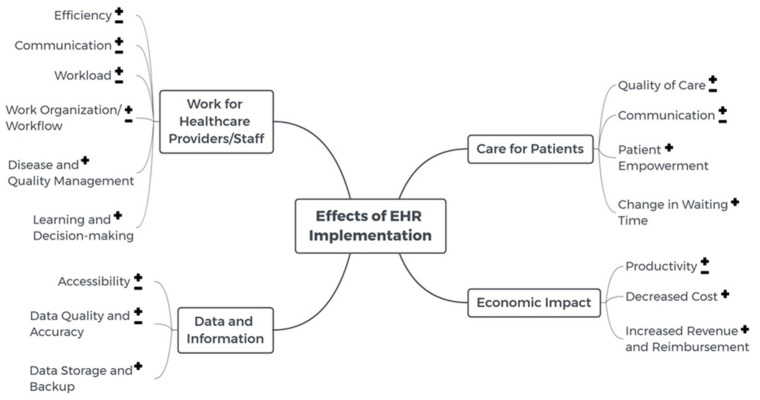
Mind map showing positive (+) and negative (−) effects of electronic health record (EHR) implementation.

Both positive and negative effects related to the work of healthcare providers/staff, data and information, care of patients, and economic impact were identified in the studies, as shown in Figure 2. 

#### 3.1.1. Work for Healthcare Providers/Staff

##### Efficiency

Improved efficiency following EHR implementation was suggested, with clinicians finding frequently used EHR functions useful for improving work efficiency [48]. Perceived general efficiency gains in workflow [27] and in laboratory turnaround time [29] were found, with these time-consuming tasks related to paper-based records being no longer required [29,41,44]. One study showed that EHR adoption did not significantly change the amount of time specialist physicians spent with each patient [21]. Another time-motion observation study showed a great reduction in time spent on administrative tasks for nurses following EHR implementation [22]. Clinicians and staff also mentioned improved efficiency through the quick retrieval of information in EHRs [20,28,42,43,44,45] and a reduction in documentation time [18,34,49,59] by, for example, using EHR templates [34]. The use of templates in EHRs was mentioned as being beneficial [45] and saved time on documentation [34]. An observational study, combining data analysis of EHR usage, suggested that clinicians completed their notes sooner post-EHR implementation (mean hours to completion 10–24 h) compared to the pre-EHR period (600–1200 h) [50]. In one study [25], interesting downward trends were found in the proportion of clinicians agreeing that EHRs resulted in longer patient visits, from 68% at month 1 post-EHR to 51% at month 12 post-EHR (*p* = 0.001). Another study reported overall positive perceptions of nurses towards EHRs in perceived use, system quality, and satisfaction [124].

Inefficiency following the implementation of EHRs was mentioned. Extensive use of EHRs in all aspects of the care process resulted in providers spending more time using EHRs during work shifts [20,28]. Providers perceived that retrieving and locating necessary information in EHRs was difficult [148] and took longer than expected, which also had a negative impact on their efficiency [34,40,45]. Clinicians expressed concerns and frustration regarding the slowness of systems [91,96,113] and the time-consuming nature of patient documentation using EHRs [23,34,38,40,43,44,58,145,154], with 81.8% of the respondents (physicians) in a survey agreeing that “to document on paper is faster than on the EHR” [39]. Another survey showed that 71% of the respondents (physicians) perceived an increase in time spent on patient documentation following the implementation of EHRs [17]. Two time-motion studies suggested similar findings, with the results indicating that nurses spent a significantly increased amount of time (*p* < 0.05) and percentage of time (*p* = 0.002) on documentation after EHR implementation [22,97]. Two other studies found that significantly more physicians reported poor or marginal sufficiency of time for documentation in settings with EHRs (46.4%, as compared to 13.6% in non-EHRs setting, *p* < 0.001) [83] and 32.8% of nurses reported an insufficient amount of time for documentation [110]. A systematic literature review concluded that compared with settings without EHR, the overall proportion of staff time spent on documentation was higher for clinicians in the presence of an EHR; for nurses in particular, the difference was statistically significant [117]. A significant decrease in efficiency (i.e., increased surgical case turnover time) that persisted for five months was shown after the implementation of EHRs [132]. Considering the usability and functionality perspectives of EHRs, failing to include key functionalities that support the workflow of the entire care team, such as the exchange of laboratory results and medication lists and tools for chronic disease management and preventive care, led to extra steps in the workflow and reduced efficiency [29,40,44]. Other design (usability) features of EHRs, including the lack of templates and the ability to reuse existing records, as well as poorly designed interfaces, also negatively affected work efficiency in two studies [43,44]. One study reported no notable improvements in physicians’ ratings for their EHRs between the years 2010 and 2014 in Finland. Instead, the results indicated the existence of serious problems and deficiencies which considerably hindered the efficiency of EHR use [136].

##### Communication

Studies suggested improvement in communication among clinicians and healthcare teams following EHR implementation. In four studies, physicians perceived improved communication as a benefit after EHR implementation [17,27,34,43]. In a longitudinal survey [25], the proportion of clinicians that agreed that communication had improved among clinicians increased from 72% to 93% (*p* < 0.001) over time (month 1 to month 12) following EHR implementation. It was reported that instant messaging in EHRs and the increased access to patient information through EHRs, enhanced communication within the healthcare team [44,45,56]. Moreover, clinicians created “huddle sheets”, listing patients’ scheduled activities and issues using EHRs [56], or utilized functions in EHRs such as patient problem lists, to-do lists, and task assignments [38] as communication tools for the healthcare team. In a study conducted in homecare settings, clinicians reported being satisfied with team communication following EHR implementation both in the survey and in interview sessions [50]. Respondents (clinicians) interviewed in the same study [50] claimed that communication using the EHR was similar to face-to-face communication. Another study set in residential aged care settings found that respondents (nurses and aged care staff) expressed that EHR adoption facilitated communication with healthcare providers from other organizations and among staff members within the organization [42].

However, the decreased frequency of direct communication among healthcare professionals was a common complaint [34,48]. Clinicians were concerned that this would distance physicians from nurses or would even diminish the opportunity for care professionals to share relevant information face to face [34]. In one study, comments made in the follow-up interview conducted 11 months after EHR implementation revealed dissatisfaction with team communication [40]. Moreover, misconceptions of communication were observed when providers had spent time carefully documenting patient information in EHRs and had thought that the information would be communicated, only later realizing that the information had not been read by colleagues [47]. Another study suggested that current EHRs do not adequately support teamwork among oncology providers [120]. Mixed effects were reported in one study as nurses’ and physicians’ experiences on EHR appeared to vary by EHR brand and employment sector [155].

##### Workload

Clinicians and staff perceived decreased workloads, as the adoption of EHRs improved communication as well as the availability and accessibility of medical records [44]. In a study measuring clinicians’ mental workload during a trial period of EHR use, the results showed significant differences for five of the six National Aeronautics and Space Administration-Task Load Index (NASA-TLX) subscales during healthcare team conferences and for all six NASA-TLX subscales during ward rounds. The differences were in favor of the use of EHRs over paper-based records [26]. 

Poor integration of workflows involving different care professionals and poor connectivity with other healthcare organizations in EHRs could result in an increased workload for providers [29,44,141]. Double/multiple documentation in different systems or double-checks for multiple resources were required to ensure that the information was correct, communicated and/or exchanged [44,96,113,148]. Primary care physicians spent more than one-half of their workday, nearly six hours, interacting with EHRs during and after clinic hours [152]. Resource constraints, such as having limited access to EHRs or not having enough user licenses for EHRs, could also lead to clinicians having to carry out extra work such as double documenting patient information [44]. In one study, new work related to the introduction of EHRs and work or workarounds addressing EHR-related errors and limitations exacerbated the work burden for clinicians following EHR implementation [29]. Clinicians also considered additional education, training and learning related to the newly implemented EHRs as extra workload for them [38,45,112]. Weak findings were indicated in [135], as only 17% of participants agreed with the impact of increased workload post-EHR implementation, despite the majority anticipating a negative impact on workload pre-implementation. Colligan et al. assessed changes in cognitive workload during the transition to adopting a commercial EHR, with the results suggesting that the difference in average scores of the cognitive workload (NASA-TLX) for participating pediatric nurses was highly significant (*p* < 0.001) over time [54]. Compared to the average score at baseline, which was measured before the implementation of the EHR, the average scores collected at both the first and the fifth shifts after the launch and use of EHR had increased by 15% [54]. Another study found that the attending and resident physicians’ total TLX score was significantly correlated with the screen item (EHR interface design), meaning that higher ratings on the screen were associated with higher mental workload [108]. Frustration levels associated with EHRs were significantly higher for attending physicians compared with residents in the emergency department [108]. Negative effects of EHRs on work life balance/physician burnout were reported [127]. Another study suggested that 69.8% of physicians with EHRs reported EHR-related stress and the prevalence of burnout symptoms among these physicians was significantly higher (27.2%, as compared to 13.6% for those without EHRs, *p* < 0.001) [83]. In addition, 19.8% of nurses in another study reported at least one symptom of burnout [110]. Alert workload was reported to be related to two of the three dimensions of burnout, including physical fatigue (*p* = 0.02) and cognitive weariness (*p* = 0.04) [139].

##### Work Organization/Workflow

The increased organization of work after EHR implementation was raised by clinicians and staff [20,28,38,91]. The use of EHRs allowed nurses and aged care staff to rely less on memory or written notes, to check which tasks had been done and which should be carried out, and to develop better care plans [20,28,42,56]. Moreover, clinicians and staff perceived that EHRs facilitated better task delegation among them and clarified team roles for non-physicians [56]. A majority of nurses perceived that EHRs helped them in planning their work [119].

However, altered workflow emerged as a negative effect [91,112,113,115] related to some of the issues mentioned above, such as poor integration of current workflows in EHRs, poor cross-organizational connectivity in EHRs and communication ambiguity [29,44,47]. Nurses reported the difference in workflow in line with how PHR communication was handled in the same clinic [141]. Clinicians complained that the workflow was disrupted since they had to wait for patients to be triaged and assigned to physicians in EHRs or for physicians to input information in EHRs before they could complete their tasks, which resulted in patient flow being impeded [45,148]. Nurses needed to mentally integrate information in order to support clinical workflow [114]. Difficulty in following the new workflow after EHR implementation was raised by clinicians and other staff [46]. Furthermore, a mismatch between workflow and EHR functionality was observed because redesigning workflows both to support new EHR functions and to create new EHR functions to meet practice needs under current workflows were reported to be difficult [38]. Gaps between EHR design and the functionality needed in the complex inpatient environment resulted in a lack of standardized workflows [89].

##### Support Disease and Quality Management

It has been suggested that the implementation of EHRs supports disease and quality management. In one study, 80% of the interviewed physicians perceived the systematic storage of information in EHRs, which supports disease management, as a positive effect [43]. In another study, customized functions of EHRs were reported to enable more thorough and efficient disease management in chronic and preventive care at one participating primary care facility [44]. The support of quality management after EHR implementation was mentioned in studies conducted in primary care and residential aged care settings. One reason for this was the ability to collect/extract clinical indicators and monitor the work performance of staff members using EHRs [38,42]. Implementation of an asthma care pathway based on the EHR reduced variability in practice and ensured adherence to high-quality national guidelines [151].

##### Support Learning and Decision-Making

The support of peer learning following EHR implementation was reported by staff and managers in residential aged care settings [42]. One study suggested that physicians reported positive effects of EHRs in terms of providing access to up-to-date knowledge [27]. This was supported by studies which showed that a majority of respondents (clinicians) agreed on the benefits of EHRs related to the support of learning and decision-making [49,99]. A review study concluded that EHRs had potential in supporting shared decision-making during clinicians’ clinical work [105]. 

#### 3.1.2. Data and Information

##### Accessibility

Improved access to patient information and records was reported as a benefit following EHR/PHR implementation [17,34,37,45,48,56,65,69,75,90,91,99,101,128,147,150,153]. Nurses perceived that increased accessibility enhanced their job performance [20,28]. Timely access to information [119], including laboratory results, radiology images and medication history were mentioned as supporting and speeding up care processes [20,28,32,43]. Moreover, increased accessibility by allowing simultaneous access to patient records was mentioned as a benefit of adopting EHRs [20,28,42,44]. Still, in one study, the proportion of clinicians who agreed that EHRs improved access to clinical information remained stable (between 92 and 95%) from month 1 to month 12 post-EHR implementation [25]. 

However, increased accessibility was reported in studies conducted at other primary care and residential aged care facilities [38,42]. One study found that the majority of responding physicians (81%) reported improved remote access to patient records [46]. Interestingly, another study also mentioned that the increased accessibility, which allowed physicians to work outside of clinics, could be considered another benefit following EHR implementation [17]. However, accessibility could be impaired, as physicians suggested that limited information was retrievable in EHRs [43]. This was reported in a follow-up interview conducted post-EHR implementation in a study showing that clinicians had trouble locating and accessing information as a result of data silos [40]. Clinicians’ perception of ease of access to patient information decreased after switching to a commercial EHR (from 80.18 to 64.13%, *p* < 0.01) [106]. A cross-sectional questionnaire survey showed that the respondents (physicians) disagreed that it was easy to access previous notes (34.7%) or patient medication lists (32.7%) and considered it difficult to check lab results (79.2%) [39].

##### Data Quality and Accuracy

Improved data accuracy was suggested following the implementation of EHRs [30,38,48,67,119,147], with positive perceptions of EHRs enabling the capture of detailed data and improved documentation quality being reported [48]. A survey of nurses found that 87.2% of the respondents perceived that EHRs helped improve documentation [57]. Another study of nurses and aged care staff reported similar findings with 44% of interview respondents perceiving an improved quality of nursing documentation in both the format and content of records [42]. Process-related and structural elements of nursing documentation in EHRs were better than paper-based records [123]. Furthermore, in another study documentation was found to be significantly more likely (*p* < 0.01) to comply with guidelines post-EHR implementation than pre-EHR implementation [50]. Patient-generated data in PHR was mentioned as being highly valued and as contributing to more accurate data [51]. 

In another survey, dissatisfaction with the completeness and correctness of data was expressed by clinicians [40]. However, the number of comments addressing dissatisfaction with data completeness and correctness decreased by half from time one (11 months post-EHR implementation) to time two (17 months post-EHR) in follow-up interview sessions [40]. Data overflow was reported to be an issue resulting from free text fields in EHRs [48]. The quantity and quality of the contents of nursing documentation were found to be better in paper-based records than in EHRs in a study [122]. Mixed perceptions were suggested in [91] as physicians raised issues related to both incomplete records and the comprehensiveness of data and information.

##### Data Storage and Backup

EHRs allowing the systematic storage of data and information were mentioned by 80% of the interview respondents (physicians) in one study [43]. In another study, participants (nurses and aged care staff) reported the convenience of data storage, as digital records in EHRs were stored on servers with backup [42]. As far as the characteristics related to the benefits of better data storage offered by EHRs/PHRs were concerned, the participants believed that data would be less likely to be lost [150], would not deteriorate over time, would prevent unauthorized edits, and would minimize physical storage space [42,43,67]. 

#### 3.1.3. Care for Patients

##### Quality of Care

Enhanced quality of care following EHR/PHR adoption was suggested [17,24,27,43,45,46,49,57,64,67,75,84,85,107,111,112,115,128,131,133,135,146,150,153]. A randomized trial suggested a significantly lower prevalence in all-cause 30-day readmissions in patients who adopted PHR as compared to non-adopters [88]. Another study found that patients’ preventive health behaviors were significantly associated with PHR use [80]. Adoption of a comprehensive EHR was found to be associated with higher quality of care [76]. In addition, the adoption of EHRs in local health departments had a positive impact on the overall health outcomes of population health (*p* = 0.031) [63]. Being able to respond quickly to care needs, provide person-centered care and carry out better follow-up care with the use of EHRs were mentioned in association with the improvement of care quality [42]. One study showed that the proportion of clinicians who agreed that EHRs improved quality of care increased significantly (*p* < 0.001) from 63% at month 1 post-EHR implementation to 86% at month 12 post-EHR implementation [25].

Improved patient safety following EHR/PHR implementation was also mentioned [24,28,47,48,86,91,99]. Compared to EHRs with multiple vendors, a basic self-developed or single-vendor EHR was associated with a significant decrease of 19.2% in the rates of patient safety events [119]. Specific EHR features such as alerts, reminders and minimum required data entries were reported to help clinicians notice critical laboratory values, prevent errors and improve patient safety [28,46,48]. The timely use of EHRs to read patient histories was suggested as being important to ensure patient safety [47]. One study showed that physicians reported positive effects of EHRs on preventing medication-related errors [27]. In another study, similar results were found, with a significant increase in the proportion of clinicians who agreed that EHRs reduced the medication-related errors observed during the 12 months following EHR implementation (from 72 to 81%, *p* = 0.03) [25]. Yet another study investigated the impact of EHRs from the perspective of patients and found that more than one-third of the survey respondents (patients) agreed or somewhat agreed that EHRs contributed to improved medical safety [19]. However, one study compared PHR-adopting patients and non-adopters and suggested no significant effect on patient safety measures [121]. Moreover, continuity of care was raised as a related aspect [33,47]. A study involving nurses showed that they positively rated (from 1.66 to 2.56 out of 5) survey items examining continuity of care with the use of EHRs [33]. Additionally, clinicians and staff perceived that they had more time to spend with patients following EHR implementation [42]. Another study demonstrated that following the implementation of EHRs nurses were able to spend more time caring for and interacting with patients as the time devoted to direct patient care increased significantly (*p* < 0.05) by 6% [22]. Significant increased time spent on patient related interventions (e.g., providing emotional support, explaining patient conditions to patient and family, and coordinating patient care) was observed [97]. Similar findings were indicated in another study where the time resident physicians spent on direct patient care increased significantly (*p* < 0.001) from 31% pre-EHR implementation to 44% post-EHR implementation [53]. Interestingly, positive quality effects associated with the US’s Meaningful Use stage 1 and stage 2 achievement were indicated, whereas no significant quality effect from EHR adoption alone was suggested [72]. Another study showed that EHR use was associated with a better process of care measure performance, but did not improve condition-specific readmission or mortality rates [62]. A neutral impact of EHRs on the patient care process and quality was suggested in [79], as neither the number nor the severity of incidents affecting patients/patient care increased post-EHR implementation and disruptions in the patient care process initially increased but stabilized to the baseline level (pre-EHR) after six months. A literature review investigated the effects of EHRs on advance directives, written statements of end-of-life quality care preferences which can help enable a good death, and concluded that EHRs could potentially support advanced directive documentation but might also create further difficulties [74].

However, patients’ concerns about impersonal care activities such as information entry in EHRs were noticed by physicians [38]. A common perception of some clinicians was that the time spent with patients and on direct patient care activities decreased as a result of the use of EHRs [20,28,34]. Additionally, other nurses perceived that because of the loss of personalization, quality of care decreased following an EHR implementation [28]. One study pointed out that the measurement of the quality indicator for access to and the timely provision of influenza vaccine was shown to be worse in nursing homes with EHRs than in such settings without EHRs [149]. Nurses reported that patient misuse of PHR for reporting medical emergencies posed potential risks for patient safety [141]. Use of copy and paste related to EHRs was suggested to put patient safety at risk [125]. Another study showed that medication errors (medication safety reports) increased five-fold after the implementation of a new EHR system in pediatric units [94]. A comparison of outcomes showed EHR downtime-exposed patients, compared with non-exposed patients, had a significant increase in the duration of operating room time (1.10 times longer, CI 1.08–1.12, *p* < 0.001) and postoperative length of stay (1.04 times longer, CI 1.01–1.08, *p* < 0.007) [81].

##### Communication

Better communication between patients and providers was reported as a benefit following the implementation of EHRs/PHRs [32,34,38,42,44,69,86,87,102,134,146,147,153]. Physicians recognized the value of being able to share patient-centered information with patients using EHRs [34,143], while clinicians perceived that new communication channels such as messaging through EHRs should improve communication between patients and providers [45]. Clinicians perceived that PHRs could support the clarification of information for patients [64]. In addition, 72% of patients in a study believed PHRs would strengthen the provider patient relationship [132]. In yet another study, patient interview data suggested that EHRs had no negative impact on physician patient communication [90]. A mixed impact for EHRs on the physician-patient relationship and communication was also found, as physicians and patients perceived these differently [84].

However, reduced face-to-face/direct communication and less eye contact between patients and physicians were also noted during their clinical consultations, as physicians were preoccupied with entering information in EHRs [43,45]. Clinicians perceived that patients’ satisfaction might be negatively affected by the use of EHRs, owing to their preoccupation with typing and looking at the screen, as well as having computers positioned between patients and clinicians [45]. In a study investigating the impact of changing from a longstanding homegrown EHR to a vendor EHR, a significant decrease in the number of participating physicians who agreed that EHR does not interfere with the ability to have face-to-face contact with patients was observed and this trend persisted for 25 months after the implementation of the vendor EHR [137].

##### Patient Empowerment

Patients’ access to full or partial medical records increased with the adoption of PHRs integrated with EHRs [51]. Patients reported greater empowerment [153] and expressed that in being given access to their medical records, they felt more like partners with healthcare providers [32] and in control of their care [65]. Moreover, a more collaborative relationship between patients and physicians was reported following the EHR implementation when physicians and patients viewed information in records and made healthcare decisions together [37,45]. Clinicians also mentioned that PHR could give patients opportunities to quality control documented information [64].

##### Change in Time Spent

One study reported a decrease in waiting time for patients as a benefit of EHRs [19], with a trend of shorter waiting times being observed among hospitals that had implemented EHRs for a longer period [19]. Another study suggested changes in patient time spent on different activities, but no significant difference was found between patient groups in EHR settings and those in paper-based record settings [156].

#### 3.1.4. Economic Impact

##### Productivity

Better productivity was suggested after the implementation of EHRs [48,85,142]. One study showed increased productivity related to the completion of documents—from 12.38 completed notes per fulltime equivalent contribution pre-EHR implementation to 127.06 completed notes per full-time equivalent contribution post-EHR implementation [50]. Another study found that the average number of task occurrences per hour for resident physicians increased significantly from 117 to 154 (*p* < 0.01; i.e., from 1.95 to 2.56 activities per minute) following an EHR implementation [53]. Concerning productivity in surgical inpatient units, a significant positive impact of EHR use on operating room utilization and bed occupancy rates was reported [130]. 

However, decreased productivity was also reported [27,38,48]. Negative perceptions of EHR productivity outcomes and effect on practice costs were indicated by physicians [104]. One study showed a reduction in long-term practice productivity post-EHR implementation across all specialities in the ambulatory practice context [55], while another found that the average number of task occurrences per hour for attending physicians decreased significantly from 138 to 106 (*p* < 0.01; i.e., from 2.30 to 1.76 activities per minute) following an EHR implementation [53].

##### Decreased Cost

Decreased costs or economic savings after EHR adoption were reported in several areas such as administrative costs, documentation costs and nursing costs [30]. One study showed a significant decrease in the monthly costs of transcription (a decline of 74.6%; *p* < 0.001) and the monthly consumption of copy paper (a decline of 26.6%; *p* < 0.001) post-EHR implementation compared to pre-EHR implementation [36]. Another study showed that both citizens and physicians (58.1% and 62.5%, respectively) agreed, and both perceived the reduced cost of healthcare as a positive effect of EHRs [112].

##### Increased Revenue and Reimbursement

One study reported increased revenues and reimbursements after the implementation of EHRs [55]. It also argued that being paid more for seeing fewer patients could be suggested as a type of efficiency by the study findings despite the observed productivity losses of the study [55].

### 3.2. Barriers to Adoption and Use

**Figure 3:**
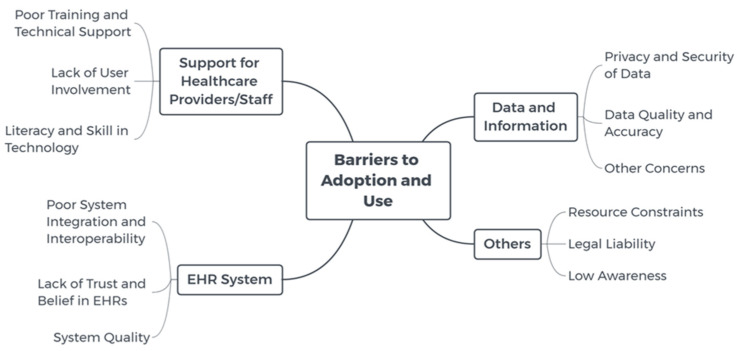
Mind map of barriers to EHR adoption and use.

Some barriers to EHR adoption and use were identified in the studies as shown in Figure 3.

#### 3.2.1. Support for End Users

Poor and insufficient training and lack of technical/educational support for users were suggested as barriers to the adoption/use of EHRs and PHRs [27,32,45,48,52,58,66,93,98,100,107,128,129,138,140,157]. Clinicians and staff considered the lack of knowledge on EHR functions to be one of the challenges faced when using EHRs [38,43]. The paucity of user involvement during the planning, development, and implementation phases of the system life cycle of EHRs and PHRs was also mentioned [32,40,48,51,66,98,138,140]. Users’ literacy, as well as skills in technology and computing, which include skills in typing [43,44] and use of the internet [32], were reported to affect EHR and PHR adoption/use [27,31,32,43,44,58,66,77,98,100,107,138,140].

#### 3.2.2. EHR/PHR System

Complaints concerning poor interoperability and integration between systems were found; these issues hindered both the implementation [48,61,93,98] and the adoption/use of EHRs and PHRs [44,47,51,56,134]. Clinicians’ resistance [86,93,123,140] and lack of trust in EHRs/PHRs [66,92,98,114] regarding issues related to data privacy and the risk of data loss, which were key challenges to fully exploiting EHRs [43], were suggested. This was highlighted in a study which showed that the survey respondents (clinicians and staff) perceived a lack of trust in the reliability of EHRs and a lack of belief in their value for patient care to be barriers to EHR implementation (mean score 3.47 out of 5 and 4.46 out of 5) [58]. Other issues regarding EHR systems such as system quality [61,100], system compatibility [138], system inefficiency (slow response) [43,48], system failures [38,39], server crashes [38], and difficulties in finding EHRs that meet needs [128] were mentioned as concerns. Functionality issues concerning EHRs included both too many complex functions [38] and too few needed functions [40,45] and were reported as barriers to their use. Usability [32] such as the design of user interface [29,32,138] and navigation [48] were agreed to be critical features. One study showed that the survey respondents (clinicians) were dissatisfied with the usability of EHRs at both months 11 and 17 post-EHR implementation (mean score 2.1 and 2.4 out of 5) [40].

#### 3.2.3. Data and Information

The privacy and security of data and information were raised as concerns by clinicians and patients for both EHRs and PHRs [27,32,35,98,100,112,129,138,140,150]. One-third of physicians in a study expressed their concern about the privacy of patient information in EHRs in cases of “illegal leakage” [43]. Regarding the use of PHRs, providers raised concerns about inappropriate and unauthorized access to the sensitive information, such as mental health information, they might contain [31,77]. It was also mentioned that patients/consumers were concerned about privacy, security and confidentiality issues related to the adoption of PHRs [32,48], while the quality of patient-generated data in PHRs was of concern to clinicians [31,35]. Concerns related to data and information in PHRs causing anxiety for patients if misinterpreted were reported [35,86,150]. Clinicians also maintained that it would be problematic if access to medical records in PHRs were to be provided to patients with psychiatric conditions [35].

#### 3.2.4. Others

Resource constraints, including cost of system upgrades/maintenance [38,93], inadequate funding [27,48,93], time constraints [48], limited access to/number of computers [39,44,47,48,157], limited networks (internet) [44,47], plus an insufficient number of user licenses [44], were reported as barriers to the implementation and adoption of EHRs/PHRs [29,77,100,128,140]. Moreover, worries about the legal liability of medical records in EHRs/PHRs were raised [43,150]. Lack of administrative and policy support [98,140], as well as low awareness which may hinder the successful adoption and use of PHRs, were mentioned [32,107,129,140]. In one study, no major barrier was identified [95].

## 4. Discussion

The goal of this scoping review was to identify currently available evidence and present an overview of the effects of EHR implementation and the barriers to EHR adoption and use. Our results, which suggest mixed findings with a predominance of positive effects and some negative effects of EHR implementation, include improved efficiency, decreased efficiency, better communication, improved accessibility and enhanced quality of care as some of the identified major effects. This is in line with a recent review which mentioned that the findings of the early literature on EHR effects on care quality, communication, and information management were notably mixed [158,159]. Despite the overall positive findings, in more recent research, mixed results and unanticipated negative consequences (e.g., disrupted workflow) were still reported [158]. Another recent systematic review focusing on EHR impact in a specific context (i.e., long-term care facilities) also suggested mixed findings but with a predominance of positive outcomes [160].

Some of the barriers with high occurrence suggested in our results are resource constraints, poor/insufficient training and a lack of technical/educational support for users, as well as poor literacy and a lack of skills in technology. Most barriers identified in this study (e.g., training and technical support, literacy and skill in technology, trust and belief in EHRs, privacy, and resources/costs) are congruent with the findings suggested by recent systematic reviews [161,162]. Interestingly, we found that many of the negative effects and barriers seemed to be consistent over time. One such factor is the impact of EHR use on clinician burnout. Recent research suggests an association between EHR use and emotional exhaustion [163], as well as between poor EHR usability and experienced time pressure and stress [66]. Another observation was that the use of definitions of EHR varied from study to study and did not comply with definitions given by ISO/TR 14639-1:2012(en) and ISO/TR 14292:2012(en). This observation, in combination with a lack of contextual information related to the study settings and systems, may hamper any application of the findings. Ammenwerth [164] points out that incomplete contextual information in publications is related to the inadequate quality of health IT evaluation publications and could make it difficult to use and generalize the evidence. After all, it is within a specific study context that an author draws conclusions about an investigated system, and it is thus the contextual information that enables readers to interpret the findings.

Efforts to improve these deficiencies could be of great value to academia, industry and society. These should be addressed by encouraging researchers and editors of scientific journals to adhere to standardized definitions [5,6], to outline the quality requirements of investigated systems based on standards [4], to clearly describe the contextual information, and to follow standardized guidelines (e.g., Statement on reporting of evaluation studies in Health Informatics (STARE-HI)) [164,165] for conducting and reporting evaluation studies.

### Limitations

This scoping review has some limitations. The fact that there is no consensus on the definition of EHR in the literature rendered the choice of search terms difficult. We could have used “electronic health record” and “personal health record” as MeSH terms (Medical Subject Headings), as they include other terms such as “electronic medical record” and “computerized medical record”. “Electronic patient record” would, however, not then have been included. As authors’ usage of MeSH terms is not stringent in all publications, we decided to look for terms in titles and abstracts instead, which resulted in more hits than using the corresponding MeSH terms. Nevertheless, the choice of search terms, with the omission of “electronic medical record”, as well as using “challenges” and “disadvantages” but omitting “barriers”, may have resulted in limited coverage of articles. Moreover, we included four different databases, which we considered to contain most publications from the medical and technical fields, resulting in the omission of other databases as well as gray literature.

Selection bias is a concern as the screening and selection process may be considered subjective. Multiple researchers’ opinions and consensus meetings were adopted in an attempt to control for this. The limited contextual information provided in the single studies and the variation in definitions of EHRs could have resulted in some relevant studies being overlooked or excluded. Moreover, the homogeneity of the origin countries of the studies (86 out of 141 from North America) and the missing contextual information made it difficult to ascertain whether there were any cultural differences and influence on the effects of an EHR implementation.

The fact that facilitators of implementation were not included in the review may be regarded a limitation, as this could help future implementation projects on how to facilitate the implementation work more than merely being aware of potential barriers.

An important limitation of the work is that a scoping review usually does not include a quality assessment of the included studies. We carried out a qualitative content analysis of the included studies to identify positive and negative effects of implementation as well as barriers to adoption without determining the quality of the individual studies. Results should therefore be considered with caution, even if we highlight effects with high occurrence in different studies, and the number of reviewed studies was relatively high. This is further impacted by the fact that individual studies that were also included in a literature review were not removed.

## 5. Conclusions

This review of the literature on the topic suggested mixed findings on the effects of EHR implementations and the ongoing barriers to EHR adoption and use. Although there appeared to be an increase in positive effects over time, some of the negative effects such as increased workload and dysfunctional workflows appeared to be stable. In addition to the fact that this review did not contain a quality analysis of the included papers, a lack of uniformity in the use of definitions of EHRs, and a lack of detailed contextual information concerning the study settings could be observed.

Researchers must follow the guidelines for the reporting of evaluation studies to enable others to compare results from different evaluation studies. This would also enable further measurement of the effects of the implementation of EHR systems and eHealth services in general.

## Figures and Tables

**Table 1:** Search strategy and the retrieved number of studies from PubMed, Web of Science, IEEE, and ACM for the two data sets.

Databases	Search Details	Number of Papers
PubMed	(“electronic health record”[TIAB] OR “EHR”[TIAB] OR “personal health record”[TIAB] OR “PHR”[TIAB] OR “patient record”[TIAB]) AND (“care pathways”[TIAB] OR workflow[TIAB] OR “work routines”[TIAB] OR workload [TIAB] OR “work process”[TIAB]) AND ((hasabstract[text] AND “loattrfree full text”[sb] AND “loattrfull text”[sb]) AND (“2005/01/01”[PDAT]: “2020/05/31”[PDAT]) AND “humans”[MeSH Terms] AND English[lang])	n = 275
	(“electronic health record”[TIAB] OR “EHR”[TIAB] OR “personal health record”[TIAB] OR “PHR”[TIAB] OR “patient record”[TIAB]) AND (efficiency[TIAB] OR advantages[TIAB] OR disadvantages[TIAB] OR satisfaction[TIAB] OR teamwork[TIAB] OR collaboration[TIAB] OR benefits[TIAB] OR challenges[TIAB]) AND ((hasabstract[text] AND “loattrfree full text”[sb] AND “loattrfull text”[sb]) AND (“2005/01/01”[PDAT]: “2020/05/31”[PDAT]) AND “humans”[MeSH Terms] AND English[lang])	n = 824
Web of Science	TOPIC: ((“electronic health record*” OR “EHR*” OR “personal health record*” OR “PHR*” OR “patient record*”) AND (“care pathways” OR workflow OR “work routines” OR workload OR “work process”)) Refined by: Web of Science categories: (medical informatics OR health care sciences services OR computer science information systems OR information science library science OR nursing OR computer science theory methods OR computer science software engineering) AND document types: (article OR review OR proceedings paper) AND languages: (English) Timespan: 2005–2020. Indexes: SCI-EXPANDED, SSCI, A&HCI, CPCI-S, CPCI-SSH, ESCI.	n = 701
	TOPIC: ((“electronic health record*” OR “EHR*” OR “personal health record*” OR “PHR*” OR “patient record*”) AND (efficiency OR advantages OR disadvantages OR satisfaction OR teamwork OR collaboration OR benefits OR challenges)) Refined by: Web of Science categories: (medical informatics OR health care sciences services OR computer science information systems OR computer science theory methods OR information science library science OR nursing OR computer science software engineering) AND languages: (English) Timespan: 2005–2020. Indexes: SCI-EXPANDED, SSCI, A&HCI, CPCI-S, CPCI-SSH, ESCI.	n = 4146
IEEE	((“electronic health record*” OR “EHR*” OR “personal health record*” OR “PHR*” OR “patient record*”) AND (“care pathways” OR workflow OR “work routines” OR workload OR “work process”))	n = 111
	((“electronic health record*” OR “EHR*” OR “personal health record*” OR “PHR*” OR “patient record*”) AND (efficiency OR advantages OR disadvantages OR satisfaction OR teamwork OR collaboration OR benefits OR challenges))	n = 1748
ACM	(“electronic health record*” OR “EHR*” OR “personal health record*” OR “PHR*” OR “patient record*”) AND (“care pathways” OR workflow OR “work routines” OR workload OR “work process”) Articles available from 2005–2020	n = 33
	(“electronic health record*” OR “EHR*” OR “personal health record*” OR “PHR*” OR “patient record*”) AND (efficiency OR advantages OR disadvantages OR satisfaction OR teamwork OR collaboration OR benefits OR challenges) Articles available from 2005–2020	n = 276

**Table 2:** General characteristics of the selected studies.

Characteristic	Number of Studies	Reference
**Year**		
2005–2007	5	[17,18,19,20,21]
2008–2010	10	[22,23,24,25,26,27,28,29,30,31]
2011–2013	14	[32,33,34,35,36,37,38,39,40,41,42,43,44,45]
2014–2016	16	[46,47,48,49,50,51,52,53,54,55,56,57,58,59,60,61]
2017–2019	91	[62,63,64,65,66,67,68,69,70,71,72,73,74,75,76,77,78,79,80,81,82,83,84,85,86,87,88,89,90,91,92,93,94,95,96,97,98,99,100,101,102,103,104,105,106,107,108,109,110,111,112,113,114,115,116,117,118,119,120,121,122,123,124,125,126,127,128,129,130,131,132,133,134,135,136,137,138,139,140,141,142,143,144,145,146,147,148,149,150,151,152]
2020 (until end of May)	5	[153,154,155,156,157]
**Country of origin**		
Australia	5	[42,77,107,123,154]
Brazil	1	[39]
Canada	5	[35,69,85,96,111]
Denmark	1	[26]
Finland	4	[47,66,136,155]
France	1	[130]
Germany	1	[150]
Greece	1	[112]
Italy	1	[52]
Japan	1	[19]
Jordan	4	[67,93,122,124]
Lebanon	1	[90]
Macao	1	[43]
Netherlands	4	[24,78,79,148]
New Zealand	1	[133]
Norway	2	[68,153]
Saudi Arabia	4	[91,92,95,156]
Singapore	1	[99]
Spain	1	[33]
Sweden	1	[64]
Thailand	1	[119]
Turkey	1	[49]
UAE	1	[58]
UK	2	[41,126]
USA	81	[17,20,21,22,23,25,27,28,29,31,34,36,37,38,40,44,45,46,50,51,53,54,55,56,57,59,60,61,62,63,65,70,71,72,73,75,76,80,81,82,83,84,87,88,89,94,97,101,103,104,106,108,109,110,113,114,115,116,118,120,121,127,128,131,132,134,135,137,138,139,141,142,143,144,145,146,147,149,151,152,157]
**Type/methodology**		
Focus group	10	[31,34,70,96,126,134,138,139,141,150]
Interview	33	[20,26,28,29,35,37,38,40,41,42,43,44,45,50,51,56,59,64,67,69,70,77,89,90,114,115,120,123,134,143,146,148,157]
Literature review	15	[18,30,32,48,74,85,86,98,100,102,105,117,125,129,140]
Measurement/analysis of EHR usage and/or other data	27	[36,40,50,55,62,63,71,72,73,76,79,80,81,82,94,103,109,113,118,121,122,130,132,144,147,149,151]
Measurement of mental workload	3	[26,54,108]
Observation	16	[20,26,28,38,40,44,47,50,59,70,71,89,108,111,148,152]
Questionnaire/survey	63	[19,20,23,24,25,27,28,33,38,39,40,46,49,50,51,54,57,58,59,60,61,64,65,66,68,71,75,78,83,84,87,89,91,92,93,95,96,99,101,104,106,107,108,110,112,119,124,126,127,128,131,133,134,135,136,137,139,142,145,146,149,153,155]
Time-motion observation	7	[17,21,22,53,97,154,156]
Randomized trial	2	[88,116]
**Participants**		
Physicians	61	[17,21,23,25,26,27,29,31,34,35,37,38,39,40,43,44,45,46,47,49,51,53,56,58,59,60,64,68,70,77,78,83,84,91,92,95,99,101,104,106,107,108,111,112,115,120,126,127,128,131,135,136,137,138,143,145,146,148,150,152,155]
Patients/citizens	23	[19,21,44,47,65,69,75,80,81,84,87,88,90,101,107,112,121,131,133,150,153,156,157]
Registered nurses or nurse practitioners	46	[20,22,23,24,25,26,28,29,33,38,40,41,42,44,47,49,50,56,57,58,59,61,64,66,67,76,78,93,95,96,97,107,110,113,114,119,124,126,135,138,141,148,149,150,154,155]
Other clinicians (e.g., physician assistant, nursing assistant, midwife, lab staff, therapist etc.)	24	[23,24,25,29,38,40,42,45,49,50,56,58,64,101,106,107,116,123,126,131,135,138,139,150]
Non-clinicians (e.g., research assistant, administrative staff, personal care worker, manager, IT staff, quality improvement staff etc.)	18	[21,29,38,40,41,42,44,50,51,56,59,77,78,95,107,126,135,142]

**Table 3:** Main themes, categories and sub-categories identified through the analysis of the studies.

Theme	Category	Sub-Category	Reference
Positive effects			
	Work for the healthcare providers		
		Better efficiency	[18,20,22,25,27,28,29,34,41,42,43,44,45,48,49,50,59,66,67,71,85,109,112,115,116,147,151]
		Better communication	[17,25,27,34,38,42,43,44,45,50,56,85,110,134,155]
		More organized at work	[20,28,38,42,56,91,110,119]
		Decreased workload	[26,44]
		Support disease and quality management	[38,42,43,44,151]
		Support learning and decision-making	[27,42,49,85,99,105]
	Data and information		
		Increased accessibility	[17,20,25,28,32,34,37,38,42,43,44,45,46,48,56,65,69,75,90,91,99,101,119,128,147,150,153]
		Data quality and accuracy	[30,38,42,48,50,51,57,67,78,91,119,122,147]
		Better data storage and backup	[42,43,67,150]
	Care for patients		
		Quality of care	[17,19,22,24,25,27,28,33,42,43,45,46,47,48,49,53,57,62,63,64,67,72,74,75,76,80,84,85,86,88,91,97,99,106,107,110,111,112,115,116,118,125,128,131,133,135,146,150,153]
		Better communication	[32,34,38,42,44,45,64,69,84,86,102,106,131,134,143,146,147,153]
		Patient empowerment	[32,37,45,51,64,65,69,86,153]
		Change in time spent for patients	[19,116,156]
	Economic impact		
		Better productivity	[48,50,53,85,130,142]
		Decreased cost	[30,36,112,151]
		Increased revenue and reimbursement	[55]
Negative effects			
	Work for the healthcare providers		
		Worse efficiency	[17,20,22,23,25,28,29,34,38,39,40,43,44,45,58,66,78,83,91,96,97,101,109,110,113,117,126,131,132,136,137,143,144,145,148,154]
		Increased workload	[29,38,44,45,54,68,83,91,96,108,110,112,113,127,135,139,141,146,148,152]
		Poor communication	[34,40,47,48,70,78,120]
		Dysfunctional workflow	[29,38,44,45,47,61,73,89,91,112,113,114,115,141,148]
	Data and information		
		Data quality and accuracy	[40,48,78,91,122,137,145]
		Decreased accessibility	[39,40,43,70,106,126]
	Care for patients		
		Face-to-face or direct communication	[43,45,84,137]
		Quality of care	[20,28,34,38,81,82,94,106,116,126,137,141,149]
	Economic impact		
		Worse productivity	[27,38,48,53,55,104]
Barriers			
	Support for users		
		Poor training and technical support	[27,32,38,43,45,48,52,58,66,93,98,100,107,128,129,138,140,157]
		Lack of user involvement	[32,40,48,51,66,98,138,140]
		Literacy and skill in technology	[27,31,32,43,44,58,66,77,98,100,107,138,140]
	EHR/PHR system		
		Poor system integration and interoperability	[44,47,48,51,56,61,93,98,133,138,140]
		Lack of trust and belief in EHRs/PHRs	[43,58,66,86,92,93,98,114,123,138,140]
		System quality	[29,32,38,39,40,43,45,48,61,77,98,100,128,138]
	Data and information		
		Privacy and security of data	[27,31,32,35,43,48,77,93,98,100,112,129,138,140,150]
		Data quality and accuracy	[31,35,77,100,107,138]
		Other concerns	[35,56]
	Others		
		Resource constraints	[27,29,38,39,44,47,48,77,93,100,128,140,157]
		Legal liability	[43,150]
		Low awareness	[32,107,129,140]
		Lack of policy support	[98,140]
		No major barrier	[95]

## References

[1] Institute of Medicine—Committee on Improving the Patient Record (1997). The Computer-Based Patient Record an Essential Technology for Health Care.

[2] Evans R.S. (2016). Electronic Health Records: Then, Now, and in the Future. Yearb. Med. Inform..

[3] Ornstein S.M., Oates R.B., Fox G.N. (1992). The computer-based medical record: Current status. J. Fam. Pract..

[4] Hoerbst A., Ammenwerth E. (2010). Electronic health records. A systematic review on quality requirements. Methods Inf. Med..

[5] International Organization for Standardization (2012). Health Informatics—Capacity-Based eHealth Architecture Roadmap—Part 1: Overview of National eHealth Initiatives.

[6] International Organization for Standardization (2012). Health Informatics—Personal Health Records—Definition, Scope and Context.

[7] Burns J. (1993). Product specification: The fifth discipline of health care. Manag. Care Q..

[8] Safran C., Bloomrosen M., Hammond W.E., Labkoff S., Markel-Fox S., Tang P.C., Detmer D.E. (2007). Toward a National Framework for the Secondary Use of Health Data: An American Medical Informatics Association White Paper. J. Am. Med. Inform. Assoc..

[9] Orfanidis L., Bamidis P.D., Eaglestone B. (2004). Data Quality Issues in Electronic Health Records: An Adaptation Framework for the Greek Health System. Health Inform. J..

[10] Voss W.G. (2016). European Union Data Privacy Law Reform: General Data Protection Regulation, Privacy Shield, and the Right to Delisting. Bus. Lawyer.

[11] Voss W.G. (2014). Looking at European Union Data Protection Law Reform through a Different Prism: The Proposed EU General Data Protection Regulation Two Years Later. J. Internet Law.

[12] McDonald C.J., Martin D.K., Overhage J.M. (1991). Standards for the electronic transfer of clinical data: Progress and promises. Top. Health Rec. Manag..

[13] Rathert C., Porter T.H., Mittler J.N., Fleig-Palmer M. (2019). Seven years after Meaningful Use: Physicians’ and nurses’ experiences with electronic health records. Health Care Manag. Rev..

[14] Hyppönen H., Kangas M., Reponen J., Nøhr C., Villumsen S., Koch S., Hardardottir G.A., Gilstad H., Jerlvall L., Pehrsson T. (2015). Nordic eHealth Benchmarking.

[15] Peters M.D., Godfrey C.M., Khalil H., McInerney P., Parker D., Soares C.B. (2015). Guidance for conducting systematic scoping reviews. Int. J. Evid. Based Healthc..

[16] Vaismoradi M., Turunen H., Bondas T. (2013). Content analysis and thematic analysis: Implications for conducting a qualitative descriptive study. Nurs. Health Sci..

[17] Pizziferri L., Kittler A.F., Volk L.A., Honour M.M., Gupta S., Wang S., Wang T., Lippincott M., Li Q., Bates D.W. (2005). Primary care physician time utilization before and after implementation of an electronic health record: A time-motion study. J. Biomed. Inform..

[18] Poissant L., Pereira J., Tamblyn R., Kawasumi Y. (2005). The impact of electronic health records on time efficiency of physicians and nurses: A systematic review. J. Am. Med. Inform. Assoc..

[19] Koide D., Asonuma M., Naito K., Igawa S., Shimizu S., Park H.A., Murray P., Delaney C. (2006). Evaluation of electronic health records from viewpoint of patients. Consumer-Centered Computer-Supported Care for Healthy People.

[20] Kossman S.P., Park H.A., Murray P., Delaney C. (2006). Perceptions of impact of electronic health records on nurses’ work. Consumer-Centered Computer-Supported Care for Healthy People.

[21] Lo H.G., Newmark L.P., Yoon C., Volk L.A., Carlson V.L., Kittler A.F., Lippincott M., Wang T., Bates D.W. (2007). Electronic health records in specialty care: A time-motion study. J. Am. Med. Inform. Assoc..

[22] Banner L., Olney C.M. (2009). Automated Clinical Documentation Does It Allow Nurses More Time for Patient Care?. CIN Comput. Inform. Nurs..

[23] Bloom M.V., Huntington M.K. (2010). Faculty, resident, and clinic staff’s evaluation of the effects of EHR implementation. Fam. Med..

[24] de Veer A.J.E., Francke A.L. (2010). Attitudes of nursing staff towards electronic patient records: A questionnaire survey. Int. J. Nurs. Stud..

[25] El-Kareh R., Gandhi T.K., Poon E.G., Newmark L.P., Ungar J., Lipsitz S., Sequist T.D. (2009). Trends in Primary Care Clinician Perceptions of a New Electronic Health Record. J. Gen. Intern. Med..

[26] Hertzum M., Simonsen J. (2008). Positive effects of electronic patient records on three clinical activities. Int. J. Med. Inform..

[27] Jha A.K., Bates D.W., Jenter C., Orav E.J., Zheng J., Cleary P., Simon S.R. (2009). Electronic health records: Use, barriers and satisfaction among physicians who care for black and Hispanic patients. J. Eval. Clin. Pract..

[28] Kossman S.P., Scheidenhelm S.L. (2008). Nurses’ perceptions of the impact of electronic health records on work and patient outcomes. CIN Comput. Inform. Nurs..

[29] McAlearney A.S., Robbins J., Hirsch A., Jorina M., Harrop J.P. (2010). Perceived efficiency impacts following electronic health record implementation: An exploratory study of an urban community health center network. Int. J. Med. Inform..

[30] Uslu A.M., Stausberg J. (2008). Value of the electronic patient record: An analysis of the literature. J. Biomed. Inform..

[31] Witry M.J., Doucette W.R., Daly J.M., Levy B.T., Chrischilles E.A. (2010). Family physician perceptions of personal health records. Perspect. Health Inf. Manag..

[32] Archer N., Fevrier-Thomas U., Lokker C., McKibbon K.A., Straus S.E. (2011). Personal health records: A scoping review. J. Am. Med. Inform. Assoc..

[33] Galimany-Masclans J., Garrido-Aguilar E., Girbau-Garcia M.R., Lluch-Canut T., Fabrellas-Padres N. (2011). New Technologies and Nursing: Use and Perception of Primary Healthcare Nurses about Electronic Health Record in Catalonia, Spain. Telemed. e-Health.

[34] Grabenbauer L., Skinner A., Windle J. (2011). Electronic Health Record Adoption—Maybe It’s not about the Money Physician Super-Users, Electronic Health Records and Patient Care. Appl. Clin. Inform..

[35] Yau G.L., Williams A.S., Brown J.B. (2011). Family physicians’ perspectives on personal health records: Qualitative study. Can. Fam. Physician.

[36] Zlabek J.A., Wickus J.W., Mathiason M.A. (2011). Early cost and safety benefits of an inpatient electronic health record. J. Am. Med. Inform. Assoc..

[37] Doyle R.J., Wang N., Anthony D., Borkan J., Shield R.R., Goldman R.E. (2012). Computers in the examination room and the electronic health record: Physicians’ perceived impact on clinical encounters before and after full installation and implementation. Fam. Pract..

[38] Goetz Goldberg D., Kuzel A.J., Feng L.B., DeShazo J.P., Love L.E. (2012). EHRs in primary care practices: Benefits, challenges, and successful strategies. Am. J. Manag. Care.

[39] Holanda A.A., Sa H., Vieira A., Catrib A.M.F. (2012). Use and Satisfaction with Electronic Health Record by Primary Care Physicians in a Health District in Brazil. J. Med. Syst..

[40] Sockolow P.S., Bowles K.H., Lehmann H.P., Abbott P.A., Weiner J.P. (2012). Community-Based, Interdisciplinary Geriatric Care Team Satisfaction with an Electronic Health Record A Multimethod Study. CIN Comput. Inform. Nurs..

[41] Waterson P., Glenn Y., Eason K. (2012). Preparing the ground for the ‘paperless hospital’: A case study of medical records management in a UK outpatient services department. Int. J. Med. Inform..

[42] Zhang Y.T., Yu P., Shen J. (2012). The benefits of introducing electronic health records in residential aged care facilities: A multiple case study. Int. J. Med. Inform..

[43] Chao W.C., Hu H., Ung C.O.L., Cai Y. (2013). Benefits and Challenges of Electronic Health Record System on Stakeholders: A Qualitative Study of Outpatient Physicians. J. Med. Syst..

[44] Howard J., Clark E.C., Friedman A., Crosson J.C., Pellerano M., Crabtree B.F., Karsh B.T., Jaen C.R., Bell D.S., Cohen D.J. (2013). Electronic health record impact on work burden in small, unaffiliated, community-based primary care practices. J. Gen. Intern. Med..

[45] Noblin A., Cortelyou-Ward K., Cantiello J., Breyer T., Oliveira L., Dangiolo M., Cannarozzi M., Yeung T., Berman S. (2013). EHR Implementation in a New Clinic: A Case Study of Clinician Perceptions. J. Med. Syst..

[46] King J., Patel V., Jamoom E.W., Furukawa M.F. (2014). Clinical benefits of electronic health record use: National findings. Health Serv. Res..

[47] Laitinen H., Kaunonen M., Astedt-Kurki P. (2014). The impact of using electronic patient records on practices of reading and writing. Health Inform. J..

[48] Nguyen L., Bellucci E., Nguyen L.T. (2014). Electronic health records implementation: An evaluation of information system impact and contingency factors. Int. J. Med. Inform..

[49] Secginli S., Erdogan S., Monsen K.A. (2014). Attitudes of health professionals towards electronic health records in primary health care settings: A questionnaire survey. Inf. Health Soc. Care.

[50] Sockolow P.S., Bowles K.H., Adelsberger M.C., Chittams J.L., Liao C. (2014). Impact of homecare electronic health record on timeliness of clinical documentation, reimbursement, and patient outcomes. Appl. Clin. Inform..

[51] Wells S., Rozenblum R., Park A., Dunn M., Bates D.W. (2014). Personal Health Records for Patients with Chronic Disease. Appl. Clin. Inform..

[52] Barbarito F., Pinciroli F., Barone A., Pizzo F., Ranza R., Mason J., Mazzola L., Bonacina S., Marceglia S. (2015). Implementing the lifelong personal health record in a regionalised health information system: The case of Lombardy, Italy. Comput. Biol. Med..

[53] Carayon P., Wetterneck T.B., Alyousef B., Brown R.L., Cartmill R.S., McGuire K., Hoonakker P.L., Slagle J., Van Roy K.S., Walker J.M. (2015). Impact of electronic health record technology on the work and workflow of physicians in the intensive care unit. Int. J. Med. Inform..

[54] Colligan L., Potts H.W.W., Finn C.T., Sinkin R.A. (2015). Cognitive workload changes for nurses transitioning from a legacy system with paper documentation to a commercial electronic health record. Int. J. Med. Inform..

[55] Howley M.J., Chou E.Y., Hansen N., Dalrymple P.W. (2015). The long-term financial impact of electronic health record implementation. J. Am. Med. Inform. Assoc..

[56] O’Malley A.S., Draper K., Gourevitch R., Cross D.A., Scholle S.H. (2015). Electronic health records and support for primary care teamwork. J. Am. Med. Inform. Assoc..

[57] Yontz L.S., Zinn J.L., Schumacher E.J. (2015). Perioperative Nurses’ Attitudes toward the Electronic Health Record. J. Perianesthesia Nurs..

[58] Bani-issa W., Al Yateem N., Al Makhzoomy I.K., Ibrahim A. (2016). Satisfaction of health-care providers with electronic health records and perceived barriers to its implementation in the United Arab Emirates. Int. J. Nurs. Pract..

[59] Chao C.A. (2016). The impact of electronic health records on collaborative work routines: A narrative network analysis. Int. J. Med. Inform..

[60] Unni P., Staes C., Weeks H., Kramer H., Borbolla D., Slager S., Taft T., Chidambaram V., Weir C. (2016). Why aren’t they happy? An analysis of end-user satisfaction with Electronic health records. AMIA Annu. Symp. Proc..

[61] Topaz M., Ronquillo C., Peltonen L.M., Pruinelli L., Sarmiento R.F., Badger M.K., Ali S., Lewis A., Georgsson M., Jeon E. (2016). Nurse Informaticians Report Low Satisfaction and Multi-level Concerns with Electronic Health Records: Results from an International Survey. AMIA Annu. Symp. Proc..

[62] Yuan N., Dudley R.A., Boscardin W.J., Lin G.A. (2019). Electronic health records systems and hospital clinical performance: A study of nationwide hospital data. J. Am. Med. Inform. Assoc..

[63] Yeung T. (2019). Local health department adoption of electronic health records and health information exchanges and its impact on population health. Int. J. Med. Inform..

[64] Wass S., Vimarlund V. (2019). Same, same but different: Perceptions of patients’ online access to electronic health records among healthcare professionals. Health Inform. J..

[65] Walker J., Leveille S., Bell S., Chimowitz H., Dong Z., Elmore J.G., Fernandez L., Fossa A., Gerard M., Fitzgerald P. (2019). OpenNotes After 7 Years: Patient Experiences with Ongoing Access to Their Clinicians’ Outpatient Visit Notes. J. Med. Internet Res..

[66] Vehko T., Hyppönen H., Puttonen S., Kujala S., Ketola E., Tuukkanen J., Aalto A.M., Heponiemi T. (2019). Experienced time pressure and stress: Electronic health records usability and information technology competence play a role. BMC Med. Inform. Decis. Mak..

[67] Tubaishat A. (2019). The effect of electronic health records on patient safety: A qualitative exploratory study. Inform. Health Soc. Care.

[68] Schopf T.R., Nedrebø B., Hufthammer K.O., Daphu I.K., Lærum H. (2019). How well is the electronic health record supporting the clinical tasks of hospital physicians? A survey of physicians at three Norwegian hospitals. BMC Health Serv. Res..

[69] Robinson S., Reed M., Quevillon T., Hirvi E. (2019). Patient perceptions and interactions with their web portal-based laboratory results. BMJ Health Care Inform..

[70] Quinn M., Forman J., Harrod M., Winter S., Fowler K.E., Krein S.L., Gupta A., Saint S., Singh H., Chopra V. (2019). Electronic health records, communication, and data sharing: Challenges and opportunities for improving the diagnostic process. Diagnosis.

[71] Pyron L., Carter-Templeton H. (2019). Improved Patient Flow and Provider Efficiency after the Implementation of an Electronic Health Record. CIN Comput. Inform. Nurs..

[72] Lin Y.K., Lin M.F., Chen H.C. (2019). Do Electronic Health Records Affect Quality of Care? Evidence from the HITECH Act. Inf. Syst. Res..

[73] Li R.C., Wang J.K., Sharp C., Chen J.H. (2019). When order sets do not align with clinician workflow: Assessing practice patterns in the electronic health record. BMJ Qual. Saf..

[74] Lemon C., De Ridder M., Khadra M. (2019). Do Electronic Medical Records Improve Advance Directive Documentation? A Systematic Review. Am. J. Hosp. Palliat. Med..

[75] Legler A., Price M., Parikh M., Nebeker J.R., Ward M.C., Wedemeyer L., Pizer S.D. (2019). Effect on VA Patient Satisfaction of Provider’s Use of an Integrated Viewer of Multiple Electronic Health Records. J. Gen. Intern. Med..

[76] Kutney-Lee A., Sloane D.M., Bowles K.H., Burns L.R., Aiken L.H. (2019). Electronic Health Record Adoption and Nurse Reports of Usability and Quality of Care: The Role of Work Environment. Appl. Clin. Inform..

[77] Kariotis T.C., Harris K.M. (2019). Clinician perceptions of My Health Record in mental health care: Medication management and sharing mental health information. Aust. J. Prim. Health.

[78] Joukes E., de Keizerl N.F., de Bruijne M.C., Abu-Hanna A., Cornett R. (2019). Impact of Electronic versus Paper-Based Record in before EHR Implementation on Health Care Professionals’ Perceptions of EHR Use, Data Quality, and Data Reuse. Appl. Clin. Inform..

[79] Jacobs M., Boersma L.J., Swart R., Mannens R., Reymen B., Korver F., van Merode F., Dekker A. (2019). Electronic Health Record implementation in a large academic radiotherapy department: Temporarily disruptions but long-term benefits. Int. J. Med. Inform..

[80] Huang J., Chen Y., Landis J.R., Mahoney K.B. (2019). Difference between Users and Nonusers of a Patient Portal in Health Behaviors and Outcomes: Retrospective Cohort Study. J. Med. Internet Res..

[81] Harrison A.M., Siwani R., Pickering B.W., Herasevich V. (2019). Clinical impact of intraoperative electronic health record downtime on surgical patients. J. Am. Med. Inform. Assoc..

[82] Graber M.L., Siegal D., Riah H., Johnston D., Kenyon K. (2019). Electronic Health Record-Related Events in Medical Malpractice Claims. J. Patient Saf..

[83] Gardner R.L., Cooper E., Haskell J., Harris D.A., Poplau S., Kroth P.J., Linzer M. (2019). Physician stress and burnout: The impact of health information technology. J. Am. Med. Inform. Assoc..

[84] Eberts M., Capurro D. (2019). Patient and Physician Perceptions of the Impact of Electronic Health Records on the Patient-Physician Relationship. Appl. Clin. Inform..

[85] Dobrow M.J., Bytautas J.P., Tharmalingam S., Hagens S. (2019). Interoperable Electronic Health Records and Health Information Exchanges: Systematic Review. JMIR Med. Inform..

[86] Dendere R., Slade C., Burton-Jones A., Sullivan C., Staib A., Janda M. (2019). Patient Portals Facilitating Engagement with Inpatient Electronic Medical Records: A Systematic Review. J. Med. Internet Res..

[87] Dalal A.K., Dykes P., Samal L., McNally K., Mlaver E., Yoon C.S., Lipsitz S.R., Bates D.W. (2019). Potential of an Electronic Health Record-Integrated Patient Portal for Improving Care Plan Concordance during Acute Care. Appl. Clin. Inform..

[88] Creber R.M.M., Grossman L.V., Ryan B., Qian M., Polubriaginof F.C.G., Restaino S., Bakken S., Hripcsak G., Vawdrey D.K. (2019). Engaging hospitalized patients with personalized health information: A randomized trial of an inpatient portal. J. Am. Med. Inform. Assoc..

[89] Assis-Hassid S., Grosz B.J., Zimlichman E., Rozenblum R., Bates D.W. (2019). Assessing EHR use during hospital morning rounds: A multi-faceted study. PLoS ONE.

[90] Antoun J., Hamadeh G., Romani M. (2019). Effect of computer use on physician-patient communication using interviews: A patient perspective. Int. J. Med. Inform..

[91] Alsohime F., Temsah M.H., Al-Eyadhy A., Bashiri F.A., Househ M., Jamal A., Hasan G., Alhaboob A.A., Alabdulhafid M., Amer Y.S. (2019). Satisfaction and perceived usefulness with newly-implemented Electronic Health Records System among pediatricians at a university hospital. Comp. Methods Prog. Biomed..

[92] Al-Rayes S.A., Alumran A., AlFayez W. (2019). The Adoption of the Electronic Health Record by Physicians. Methods Inf. Med..

[93] Al-Rawajfah O., Tubaishat A. (2019). Barriers and facilitators to using electronic healthcare records in Jordanian hospitals from the nurses’ perspective: A national survey. Inf. Health Soc. Care.

[94] Whalen K., Lynch E., Moawad I., John T., Lozowski D., Cummings B.M. (2018). Transition to a new electronic health record and pediatric medication safety: Lessons learned in pediatrics within a large academic health system. J. Am. Med. Inform. Assoc..

[95] Taisan E.A.A., Seliaman M.E. Perceived Barriers and Drivers of Health Information Systems Adoption by Public Hospitals in Alhasa. Proceedings of the 2018 21st Saudi Computer Society National Computer Conference (NCC).

[96] Strudwick G., Hall L.M., Nagle L., Trbovich P. (2018). Acute care nurses’ perceptions of electronic health record use: A mixed method study. Nurs. Open.

[97] Schenk E., Schleyer R., Jones C.R., Fincham S., Daratha K.B., Monsen K.A. (2018). Impact of Adoption of a Comprehensive Electronic Health Record on Nursing Work and Caring Efficacy. CIN Comput. Inform. Nurs..

[98] Priestman W., Sridharan S., Vigne H., Collins R., Seamer L., Sebire N.J. (2018). What to expect from electronic patient record system implementation; lessons learned from published evidence. J. Innov. Health Inform..

[99] Osop H., Sahama T. Doctors’ perception of the potential of EHR: A Singapore insight. Proceedings of the Australasian Computer Science Week Multiconference.

[100] O’Donnell A., Kaner E., Shaw C., Haighton C. (2018). Primary care physicians’ attitudes to the adoption of electronic medical records: A systematic review and evidence synthesis using the clinical adoption framework. BMC Med. Inform. Decis. Mak..

[101] Meyerhoefer C.D., Sherer S.A., Deily M.E., Chou S.Y., Guo X.H., Chen J., Sheinberg M., Levick D. (2018). Provider and patient satisfaction with the integration of ambulatory and hospital EHR systems. J. Am. Med. Inform. Assoc..

[102] McMillan B., Eastham R., Brown B., Fitton R., Dickinson D. (2018). Primary Care Patient Records in the United Kingdom: Past, Present, and Future Research Priorities. J. Med. Internet Res..

[103] Marmor R.A., Clay B., Millen M., Savides T.J., Longhurst C.A. (2018). The Impact of Physician EHR Usage on Patient Satisfaction. Appl. Clin. Inform..

[104] Lim M.C., Boland M.V., McCannel C.A., Saini A., Chiang M.F., Epley K.D., Lum F. (2018). Adoption of Electronic Health Records and Perceptions of Financial and Clinical Outcomes among Ophthalmologists in the United States. JAMA Ophthalmol..

[105] Kuo A.M.S., Thavalathil B., Elwyn G., Nemeth Z., Dang S. (2018). The Promise of Electronic Health Records to Promote Shared Decision Making: A Narrative Review and a Look Ahead. Med. Decis. Mak..

[106] Krousel-Wood M., McCoy A.B., Ahia C., Holt E.W., Trapani D.N., Luo Q.Y., Price-Haywood E.G., Thomas E.J., Sittig D.F., Milani R.V. (2018). Implementing electronic health records (EHRs): Health care provider perceptions before and after transition from a local basic EHR to a commercial comprehensive EHR. J. Am. Med. Inform. Assoc..

[107] Khan U.R., Zia T.A., Pearce C., Perera K., Siuly S., Lee I., Huang Z., Zhou R., Wang H., Xiang W. (2018). Perceptions and Experiences of General Practice Users about My Health Record. Health Information Science.

[108] Khairat S., Burke G., Archambault H., Schwartz T., Larson J., Ratwani R.M. (2018). Perceived Burden of EHRs on Physicians at Different Stages of Their Career. Appl. Clin. Inform..

[109] Kannampallil T.G., Denton C.A., Shapiro J.S., Patel V.L. (2018). Efficiency of Emergency Physicians: Insights from an Observational Study using EHR Log Files. Appl. Clin. Inform..

[110] Harris D.A., Haskell J., Cooper E., Crouse N., Gardner R. (2018). Estimating the association between burnout and electronic health record-related stress among advanced practice registered nurses. Appl. Nurs. Res..

[111] Graham T.A.D., Ballermann M., Lang E., Bullard M.J., Parsons D., Mercuur G., San Agustin P., Ali S. (2018). Emergency Physician Use of the Alberta Netcare Portal, a Province-Wide Interoperable Electronic Health Record: Multi-Method Observational Study. JMIR Med. Inform..

[112] Entzeridou E., Markopoulou E., Mollaki V. (2018). Public and physician’s expectations and ethical concerns about electronic health record: Benefits outweigh risks except for information security. Int. J. Med. Inform..

[113] Dudding K.M., Gephart S.M., Carrington J.M. (2018). Neonatal Nurses Experience Unintended Consequences and Risks to Patient Safety with Electronic Health Records. CIN Comput. Inform. Nurs..

[114] Despins L.A., Wakefield B.J. (2018). The Role of the Electronic Medical Record in the Intensive Care Unit Nurse’s Detection of Patient Deterioration: A Qualitative Study. CIN Comput. Inform. Nurs..

[115] Denton C.A., Soni H.C., Kannampallil T.G., Serrichio A., Shapiro J.S., Traub S.J., Patel V.L. (2018). Emergency Physicians’ Perceived Influence of EHR Use on Clinical Workflow and Performance Metrics. Appl. Clin. Inform..

[116] Bruns E.J., Hook A.N., Parker E.M., Esposito I., Sather A., Parigoris R.M., Lyon A.R., Hyde K.L. (2018). Impact of a Web-Based Electronic Health Record on Behavioral Health Service Delivery for Children and Adolescents: Randomized Controlled Trial. J. Med. Internet Res..

[117] Baumann L.A., Baker J., Elshaug A.G. (2018). The impact of electronic health record systems on clinical documentation times: A systematic review. Health Policy.

[118] Bae J., Rask K.J., Becker E.R. (2018). The Impact of Electronic Medical Records on Hospital-Acquired Adverse Safety Events: Differential Effects between Single-Source and Multiple-Source Systems. Am. J. Med. Qual..

[119] Auefuea S., Nartthanarung A., Pronsawatchai P., Soontornpipit P. The Perspective of Users after the Trial of the Electronic Record System in Home Health Care Unit. Proceedings of the 2018 International Electrical Engineering Congress (iEECON).

[120] Asan O., Nattinger A.B., Gurses A.P., Tyszka J.T., Yen T.W.F. (2018). Oncologists’ Views Regarding the Role of Electronic Health Records in Care Coordination. JCO Clin. Cancer Inform..

[121] Aljabri D., Dumitrascu A., Burton M.C., White L., Khan M., Xirasagar S., Horner R., Naessens J. (2018). Patient portal adoption and use by hospitalized cancer patients: A retrospective study of its impact on adverse events, utilization, and patient satisfaction. BMC Med. Inform. Decis. Mak..

[122] Akhu-Zaheya L., Al-Maaitah R., Hani S.B. (2018). Quality of nursing documentation: Paper-based health records versus electronic-based health records. J. Clin. Nurs..

[123] Yung A. (2017). Adoption of Electronic Health Record System in Community-Based Physiotherapy Clinics: A Pilot Case Study. Stud. Health Technol. Inform..

[124] Tubaishat A. (2017). Evaluation of Electronic Health Record Implementation in Hospitals. CIN Comput. Inform. Nurs..

[125] Tsou A.Y., Lehmann C.U., Michel J., Solomon R., Possanza L., Gandhi T. (2017). Safe Practices for Copy and Paste in the EHR. Systematic Review, Recommendations, and Novel Model for Health IT Collaboration. Appl. Clin. Inform..

[126] Snowden A., Kolb H. (2017). Two years of unintended consequences: Introducing an electronic health record system in a hospice in Scotland. J. Clin. Nurs..

[127] Robertson S.L., Robinson M.D., Reid A. (2017). Electronic Health Record Effects on Work-Life Balance and Burnout within the I(3) Population Collaborative. J. Grad. Med. Educ..

[128] Raglan G.B., Margolis B., Paulus R.A., Schulkin J. (2017). Electronic Health Record Adoption among Obstetrician/Gynecologists in the United States: Physician Practices and Satisfaction. J. Healthc. Qual..

[129] Powell K.R. (2017). Patient-Perceived Facilitators of and Barriers to Electronic Portal Use: A Systematic Review. CIN Comput. Inform. Nurs..

[130] Plantier M., Havet N., Durand T., Caquot N., Amaz C., Philip I., Biron P., Perrier L. (2017). Does adoption of electronic health records improve organizational performances of hospital surgical units? Results from the French e-SI (PREPS-SIPS) study. Int. J. Med. Inform..

[131] Ochoa A., Kitayama K., Uijtdehaage S., Vermillion M., Eaton M., Carpio F., Serota M., Hochman M.E. (2017). Patient and provider perspectives on the potential value and use of a bilingual online patient portal in a Spanish-speaking safety-net population. J. Am. Med. Inform. Assoc..

[132] McDowell J., Wu A., Ehrenfeld J.M., Urman R.D. (2017). Effect of the Implementation of a New Electronic Health Record System on Surgical Case Turnover Time. J. Med. Syst..

[133] Liew C.L., Harjadinata J. Patient Portal Service: An Exploration of Patients’ Experience and Perception. Proceedings of the ICDS 2017: The Eleventh International Conference on Digital Society.

[134] King G., Maxwell J., Karmali A., Hagens S., Pinto M., Williams L., Adamson K. (2017). Connecting Families to Their Health Record and Care Team: The Use, Utility, and Impact of a Client/Family Health Portal at a Children’s Rehabilitation Hospital. J. Med. Internet Res..

[135] Kelly M.M., Dean S.M., Carayon P., Wetterneck T.B., Hoonakker P.L.T. (2017). Healthcare Team Perceptions of a Portal for Parents of Hospitalized Children Before and After Implementation. Appl. Clin. Inform..

[136] Kaipio J., Lääveri T., Hyppönen H., Vainiomäki S., Reponen J., Kushniruk A., Borycki E., Vänskä J. (2017). Usability problems do not heal by themselves: National survey on physicians’ experiences with EHRs in Finland. Int. J. Med. Inform..

[137] Hanauer D.A., Branford G.L., Greenberg G., Kileny S., Couper M.P., Zheng K., Choi S.W. (2017). Two-year longitudinal assessment of physicians’ perceptions after replacement of a longstanding homegrown electronic health record: Does a J-curve of satisfaction really exist?. J. Am. Med. Inform. Assoc..

[138] Hamamura F.D., Withy K., Hughes K. (2017). Identifying Barriers in the Use of Electronic Health Records in Hawai’i. Hawaii. J. Med. Public Health.

[139] Gregory M.E., Russo E., Singh H. (2017). Electronic Health Record Alert-Related Workload as a Predictor of Burnout in Primary Care Providers. Appl. Clin. Inform..

[140] Gesulga J.M., Berjame A., Moquiala K.S., Galido A. Barriers to Electronic Health Record System Implementation and Information Systems Resources: A Structured Review. Proceedings of the 4th Information Systems International Conference.

[141] Gerber D.E., Beg M.S., Duncan T., Gill M., Lee S.J.C. (2017). Oncology Nursing Perceptions of Patient Electronic Portal Use: A Qualitative Analysis. Oncol. Nurs. Forum.

[142] Frogner B.K., Wu X.L., Ku L., Pittman P., Masselink L.E. (2017). Do Years of Experience with Electronic Health Records Matter for Productivity in Community Health Centers?. J. Ambul. Care Manag..

[143] Fletcher K.E., Asan O., Tyszka J. (2017). Residents’ Insights and Ideas about Screen-Sharing in Primary Care Clinics. Appl. Clin. Inform..

[144] Feblowitz J., Takhar S.S., Ward M.J., Ribeira R., Landman A.B. (2017). A Custom-Developed Emergency Department Provider Electronic Documentation System Reduces Operational Efficiency. Ann. Emerg. Med..

[145] Doberne J.W., Redd T., Lattin D., Yackel T.R., Eriksson C.O., Mohan V., Gold J.A., Ash J.S., Chiang M.F. (2017). Perspectives and Uses of the Electronic Health Record Among US Pediatricians A National Survey. J. Ambul. Care Manag..

[146] Bush R.A., Connelly C.D., Perez A., Chan N., Kuelbs C., Chiang G.J. (2017). Physician Perception of the Role of the Patient Portal in Pediatric Health. J. Ambul. Care Manag..

[147] Bobadilla J.L., Roe C.S., Estes P., Lackey J., Steltenkamp C.L. (2017). Leveraging Electronic Health Record Implementation to Facilitate Clinical and Operational Quality Improvement in an Ambulatory Surgical Clinic. J. Ambul. Care Manag..

[148] Blijleven V., Koelemeijer K., Jaspers M. (2017). Identifying and eliminating inefficiencies in information system usage: A lean perspective. Int. J. Med. Inform..

[149] Bjarnadottir R.I., Herzig C.T.A., Travers J.L., Castle N.G., Stone P.W. (2017). Implementation of Electronic Health Records in US Nursing Homes. CIN Comput. Inform. Nurs..

[150] Baudendistel I., Winkler E.C., Kamradt M., Brophy S., Langst G., Eckrich F., Heinze O., Bergh B., Szecsenyi J., Ose D. (2017). Cross-sectoral cancer care: Views from patients and health care professionals regarding a personal electronic health record. Eur. J. Cancer Care.

[151] Bartlett K.W., Parente V.M., Morales V., Hauser J., McLean H.S. (2017). Improving the Efficiency of Care for Pediatric Patients Hospitalized with Asthma. Hosp. Pediatr..

[152] Arndt B.G., Beasley J.W., Watkinson M.D., Temte J.L., Tuan W.J., Sinsky C.A., Gilchrist V.J. (2017). Tethered to the EHR: Primary Care Physician Workload Assessment Using EHR Event Log Data and Time-Motion Observations. Ann. Fam. Med..

[153] Zanaboni P., Kummervold P.E., Sorensen T., Johansen M.A. (2020). Patient Use and Experience with Online Access to Electronic Health Records in Norway: Results from an Online Survey. J. Med. Internet Res..

[154] Walker R.M., Burmeister E., Jeffrey C., Birgan S., Garrahy E., Andrews J., Hada A., Aitken L.M. (2020). The impact of an integrated electronic health record on nurse time at the bedside: A pre-post continuous time and motion study. Collegian.

[155] Kaipio J., Kuusisto A., Hyppönen H., Heponiemi T., Lääveri T. (2020). Physicians’ and nurses’ experiences on EHR usability: Comparison between the professional groups by employment sector and system brand. Int. J. Med. Inform..

[156] Jabour A.M. (2020). The Impact of Electronic Health Records on the Duration of Patients’ Visits: Time and Motion Study. JMIR Med. Inform..

[157] Greysen S.R., Magan Y., Rosenthal J., Jacolbia R., Auerbach A.D., Harrison J.D. (2020). Patient Recommendations to Improve the Implementation of and Engagement with Portals in Acute Care: Hospital-Based Qualitative Study. J. Med. Internet Res..

[158] Atasoy H., Greenwood B.N., McCullough J.S. (2019). The Digitization of Patient Care: A Review of the Effects of Electronic Health Records on Health Care Quality and Utilization. Annu. Rev. Public Health.

[159] Alami H., Lehoux P., Gagnon M.-P., Fortin J.-P., Fleet R., Ag Ahmed M.A. (2020). Rethinking the electronic health record through the quadruple aim: Time to align its value with the health system. BMC Med. Inform. Decis. Mak..

[160] Kruse C.S., Mileski M., Vijaykumar A.G., Viswanathan S.V., Suskandla U., Chidambaram Y. (2017). Impact of Electronic Health Records on Long-Term Care Facilities: Systematic Review. JMIR Med. Inform..

[161] Kruse C.S., Kristof C., Jones B., Mitchell E., Martinez A. (2016). Barriers to Electronic Health Record Adoption: A Systematic Literature Review. J. Med. Syst..

[162] Kruse C.S., Kothman K., Anerobi K., Abanaka L. (2016). Adoption Factors of the Electronic Health Record: A Systematic Review. JMIR Med. Inform..

[163] Adler-Milstein J., Zhao W., Willard-Grace R., Knox M., Grumbach K. (2020). Electronic health records and burnout: Time spent on the electronic health record after hours and message volume associated with exhaustion but not with cynicism among primary care clinicians. J. Am. Med. Inform. Assoc..

[164] Ammenwerth E. (2015). Evidence-based Health Informatics: How Do We Know What We Know?. Methods Inf. Med..

[165] Talmon J., Ammenwerth E., Brender J., de Keizer N., Nykanen P., Rigby M. (2009). STARE-HI—Statement on reporting of evaluation studies in Health Informatics. Int. J. Med. Inform..

